# Subchondral rafting wires reduce tibial plateau fracture subsidence

**DOI:** 10.1007/s00590-024-03963-1

**Published:** 2024-05-09

**Authors:** Joseph T. Patterson, Daniel Rusu, Andrew M. Duong, Vivek Satish, Max Yang, Lucas Mayer, Michael Allen, Geoffrey S. Marecek

**Affiliations:** 1 Department of Orthopaedic Surgery, Keck School of Medicine, University of Southern California, 1520 San Pablo Street, Suite 2000, Los Angeles, CA 90033-5322 USA; 2 Department of Orthopaedic Surgery, Cedars Sinai Medical Center, Los Angeles, CA USA

**Keywords:** Fracture, Open reduction internal fixation, Rafting wires, Subsidence, Tibial plateau

## Abstract

**Purpose:**

To determine if subchondral rafting wires retained as adjunctive tibial plateau fracture fixation affect postoperative articular subsidence.

**Methods:**

A retrospective cohort study was conducted at one Level 1 trauma center and one academic university hospital. Consecutive adults with closed, displaced OTA/AO 41B/C tibial plateau fractures treated between 2018 and 2023 with open reduction internal fixation were included. Patients who were not ambulatory, with contralateral injuries limiting weight bearing, and without follow-up radiographs of the injured extremity were excluded. The intervention was retention of subchondral rafting wires as definitive fixation. The primary outcome was linear articular surface subsidence between postoperative and follow-up AP knee radiographs. Linear subsidence was compared between groups using Welch’s two sample *t* test. Associations of linear subsidence with patient, injury, and treatment characteristics were assessed by multivariable linear regression.

**Results:**

We identified 179 patients of a mean age of 44 ± 14 years, of whom 15 (8.4%) received subchondral rafting wires. Median follow-up was 121 days. No patients who received rafting wires as definitive implants experienced linear subsidence ≥ 2 mm, while 22 patients (13.4%) who did not receive rafting wires experienced linear subsidence ≥ 2 mm (*p* = 0.130). Subchondral rafting wires were associated with less linear subsidence (0.3 mm [95% confidence interval − 0.3–0.9 mm] vsersus 1.0 mm [− 0.9–2.9 mm], *p* < 0.001). The depth of linear subsidence was significantly associated on multivariable regression with male sex, depressed plateau area, active smoking, and retained rafting wires.

**Conclusion:**

Subchondral rafting wires were associated with a small reduction in articular subsidence after internal fixation of tibial plateau fractures. Routine rafting wires may be useful for patients and fractures at high risk of articular subsidence.

## Introduction

Tibial plateau fractures account for approximately 1% of all fractures and occur at an incidence of 10.3 per 100,000 person-years [1, 2]. Patients with a displaced tibial plateau typically benefit from surgical open reduction and internal fixation (ORIF) to restore joint alignment, knee stability, and reduce the risk or delay the onset of post-traumatic arthritis [3]. However, postoperative subsidence of the articular surface complicates internal fixation of the tibial plateau in 6–33% of patients, with greater subsidence increasing the risk of post-traumatic arthritis [4–6]. Approximately 21–58% of patients with a displaced tibial plateau fracture develop posttraumatic osteoarthritis and experience profound negative effects on long-term functional outcomes [7–10].

**Fig. 1:**
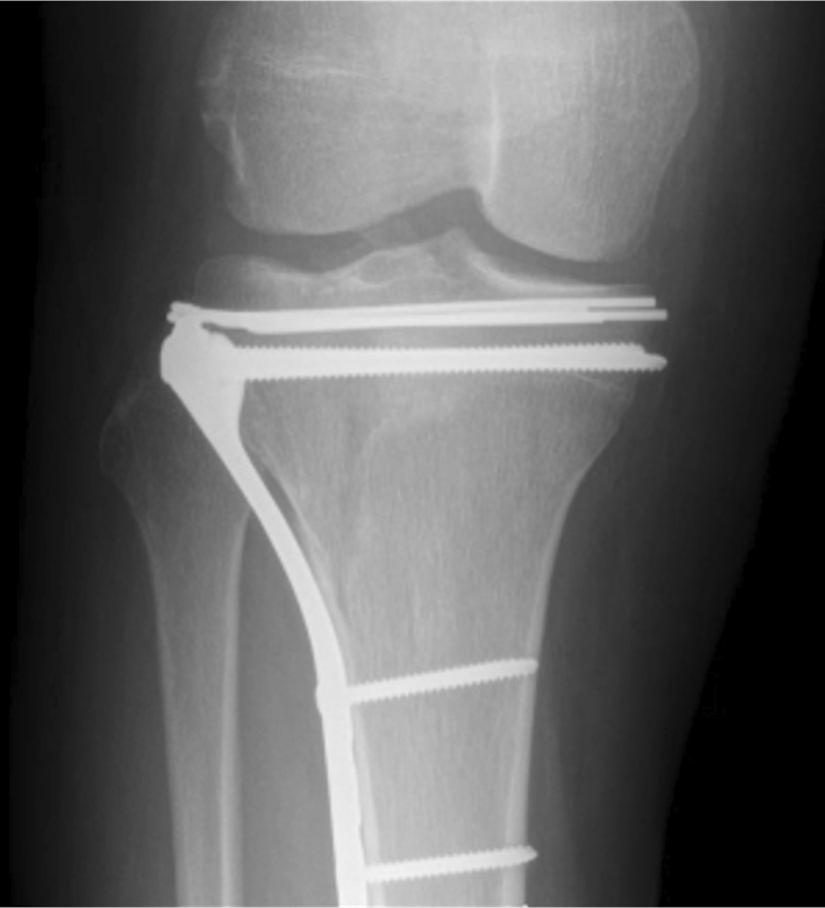
Radiographic image of inside out rafting wire technique where subchondral rafting wires are cut, bent, and retained as definitive implants

Strategies to prevent articular subsidence after tibial plateau ORIF include the use of bone graft, bone graft substitutes, and subchondral rafting fixation with screws or wires [3, 11–13]. Subchondral screws are effective for preventing subsidence [11] but may be challenging to remove for implant-associated discomfort or infection [12]. Conversely, smooth Kirschner wires or Steinmann pins are often applied to the epiphyseal or subchondral bone beneath the articular surface as provisional fixation of the fracture prior to definitive implant placement. These wires are typically removed at the conclusion of fixation. However, rafting wires may also be retained as definitive implants. Any effect of subchondral rafting wires as definitive implants (Fig. 1) on the incidence and magnitude of articular subsidence has not, to our knowledge, been established.

We investigate the effect of subchondral rafting wires as definitive implants on articular subsidence after ORIF of tibial plateau fractures. We hypothesized that use of subchondral rafting wires is associated with lower magnitude of postoperative linear subsidence of the fracture articular surface.

## Patients/methods

A retrospective cohort study was conducted at one Level 1 trauma center and one academic university hospital. Skeletally mature adults treated by one of eleven orthopaedic trauma fellowship-trained orthopaedic surgeons for closed, displaced Orthopaedic Trauma Association/ Arbeitsgemeinschaft für Osteosynthesefragen (OTA/AO) 41B or 41C [14] unicondylar or bicondylar tibial plateau fracture between 2018 and 2023 were identified from institutional surgical databases. Patients not ambulatory prior to injury or after lifting weight bearing restrictions, with contralateral injuries limiting weight bearing, or without follow-up radiographs of the injured extremity were excluded. Injury, intraoperative, and postoperative imaging were reviewed to confirm eligibility.

The intervention was retention of any smooth Kirschner wire(s) or Steinmann pin(s) in the epiphysis of the proximal tibia supporting the articular surface as a definitive internal fixation implant identified by imaging and confirmed by review of the operative report. The comparator was the absence of any rafting wire(s) as definitive fixation. Rafting wires were placed by three of the study surgeons. Single-end or double-ended wires were placed to raft the subchondral epiphyseal bone in either a retrograde or antegrade manner, as appropriate per the reduction and fixation tactics for each surgical case. At the conclusion of definitive plate and screw fixation, wires were withdrawn 1 cm, cut, bent 180°, cut, and impacted into bone per the method of Benirschke et al. [15].

**Fig. 2:**
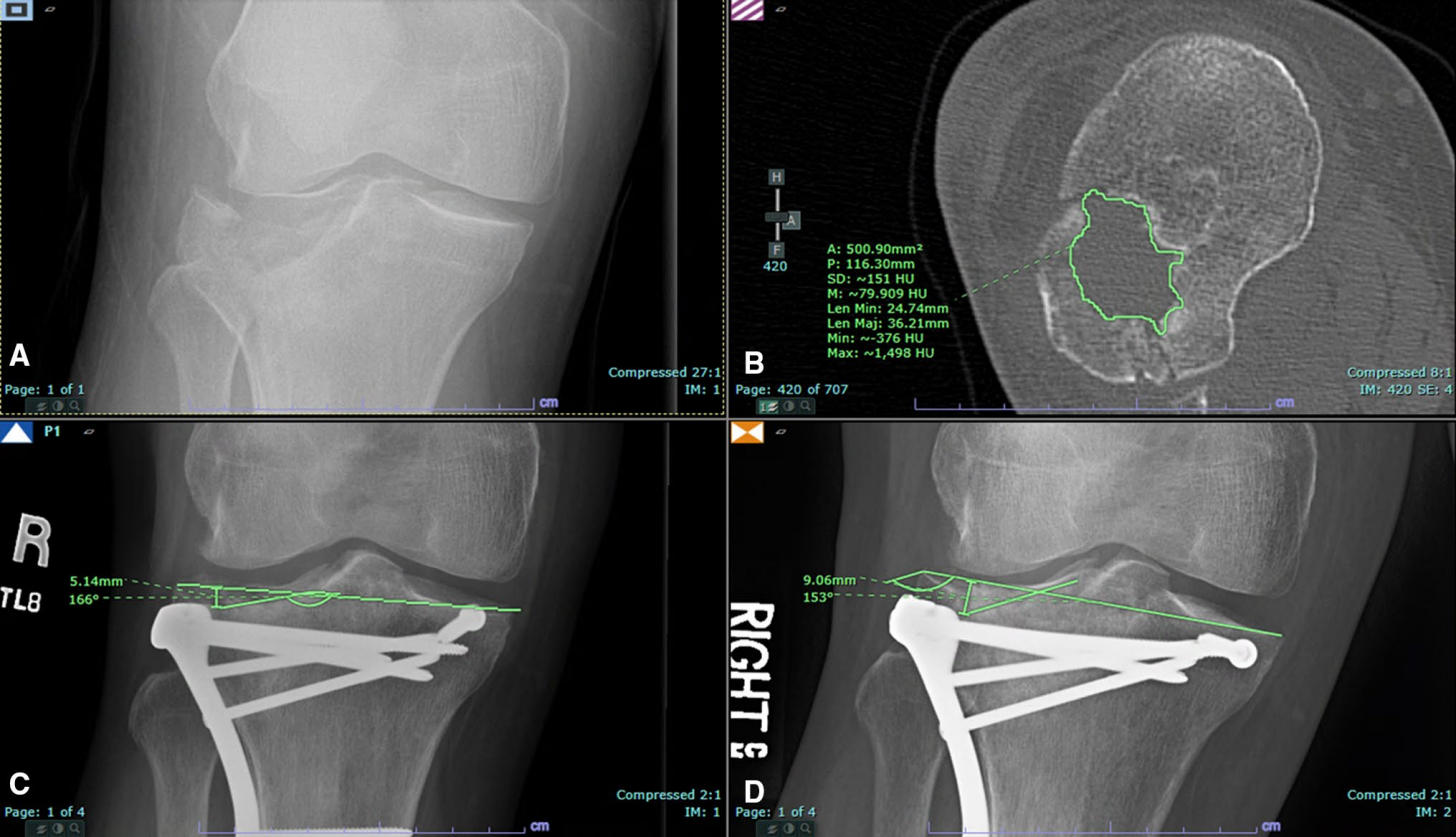
Anteroposterior (AP) radiograph of the right knee demonstrating a displaced fracture of the tibial plateau. **B** Measurement of depressed area. **C** Linear measurement of distance from most depressed fragment to tibial plateau joint line on post-operative AP radiograph. **D** Linear measurement of distance from most depressed fragment to tibial plateau joint line on AP radiograph at final follow-up demonstrating interval linear subsidence of 3.9 mm

The primary outcome measure was linear subsidence assessed as the difference in distance from the joint line to the most depressed articular fragment between immediate postoperative and final follow-up anteroposterior (AP) radiographs of the knee or tibia. The maximal plateau depression (MPD) was measured as the distance from the joint line, determined from the superior aspects of the lateral and medial plateaus, to the most depressed part of the articular surface (Fig. 2A) [5]. Linear subsidence was defined as the difference in MPD between the final and immediate postoperative follow-ups. Negative linear subsidence measurements (e.g. depressed segment found to be more cranial and proximal to joint line at follow-up versus immediately postoperatively) were considered artifactual secondary to poor radiographic technique, specifically failure to obtain the AP radiograph with tibial plateau slope. Patients with negative linear subsidence were therefore excluded. Fracture extension into the medial coronal plateau was noted and the depressed plateau area was measured in axial CT images (Fig. 2C) [5, 16]. Imaging measurements were performed independently by two investigators and averaged to reduce measurement error at both sites. Measurements were performed using Synapse V7.3.000 and V5.7.245U on dedicated radiology workstations, respectively, at each site (FUJIFILM Healthcare Americas Corporation, Lexington, MA). Each image was made full screen using the zoom feature prior to measurement.

Covariables potentially associated with intervention and outcome were collected including days to surgery, follow-up time, age [5], sex, race, ethnicity, body mass index (BMI), osteoporosis, corticosteroid use, alcohol abuse, smoking status, domiciled status, Charleston Comorbidity Index (CCI), meniscal repair, and compliance with weight bearing precautions were abstracted from a trauma registry and chart review. Fracture characteristics including AO/OTA and Schatzker classifications, the presence of a medial coronal fracture line [17], condylar widening, area of articular depression [5], and articular comminution [6], were determined from pre-operative CT scans. Fixation characteristics including screw diameter, screw number, and linear distance from the articular surface to the most proximal wire and screw [6] were assessed on postoperative radiographs.

Linear subsidence was approximated to a normal distribution by logarithmic transformation and compared between groups using Welch two sample *t* test. A multivariable linear regression model was used to assess the effect of rafting wires on linear subsidence considering potential confounding by patient, injury, and treatment characteristics. The model was optimized using Akike’s Information Criterion. Statistically significant differences were defined by *α* = 0.050. Statistical analyses were performed using RStudio V2023.06.1 + 524 (Posit Software, Boston, MA).

## Results

**Fig. 3:**
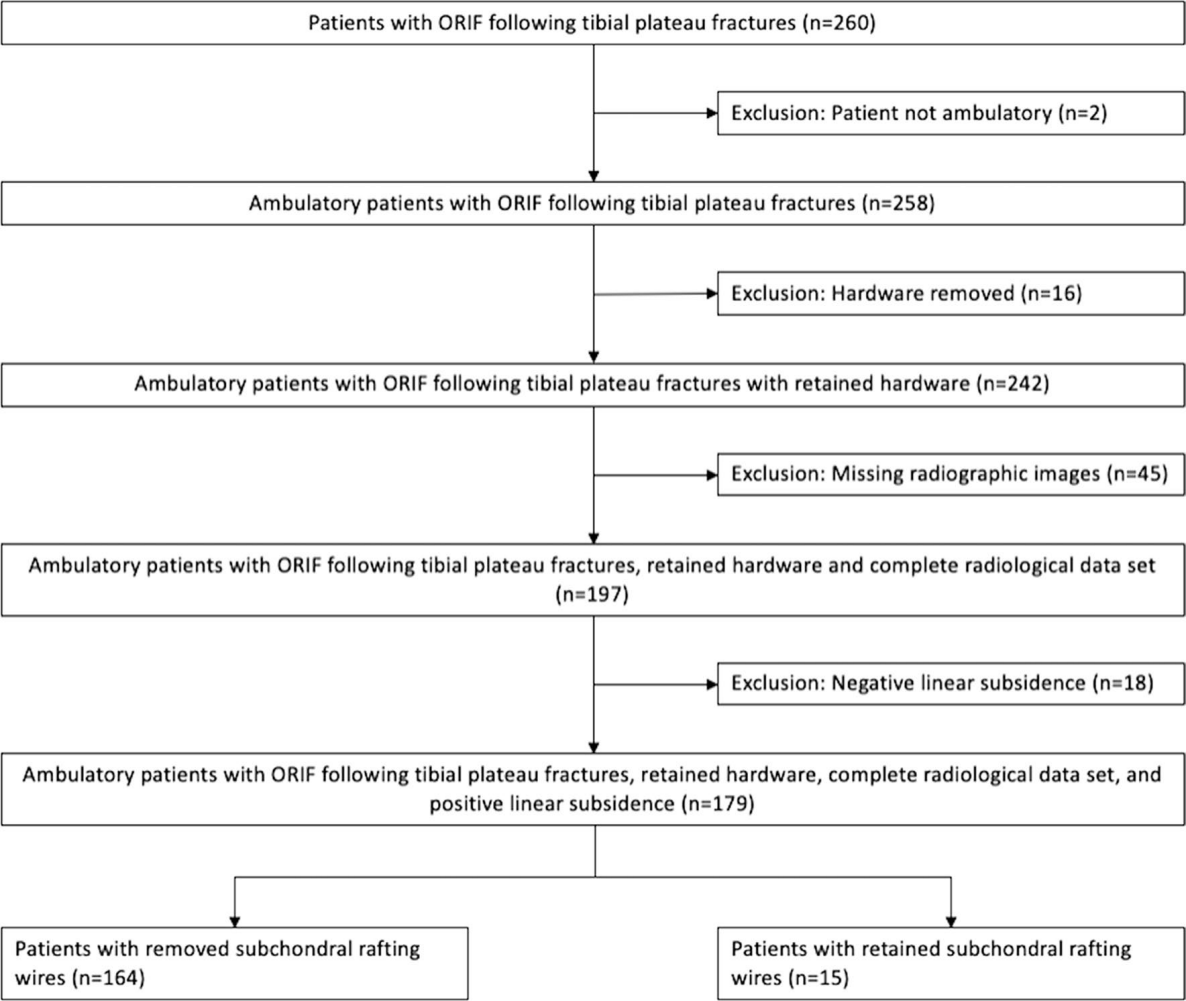
STROBE diagram

Two hundred sixty patients with tibial plateau fractures treated with ORIF were screened and 179 patients met inclusion criteria (Fig. 3). Included patients were 28% women and aged a mean 44 ± 14 years. Of these, 15 patients (8.4%) were treated with subchondral rafting wire fixation. Median follow-up was 121 days (IQR = 139 days). No significant differences by treatment were observed with regard to age, sex, race, ethnicity, body mass index, Charlson comorbidity index, osteopenia, osteoporosis, corticosteroid use, alcohol abuse, smoking, homelessness, days from injury to surgery, fracture type or pattern, meniscus repair treatment, or weight bearing compliance after surgery (Table 1).

**Table 1:** Baseline patient and injury characteristics

	Removed K-wires (*N* = 164)		Retained K-wires (*N* = 15)		*p* value
Characteristic	Mean	SD	Mean	SD	*t* test
Age (years)	44	14	43	14	0.87
Male sex	120	73.2%	9	60.0%	0.28
Race					0.24
Black or African	10	6.1%	0	–	
American					
White	16	9.8%	3	20.0%	
Asian	6	3.7%	0	–	
Multiple	6	3.7%	1	6.7%	
Other	118	72.0%	10	66.7%	
Not stated	8	4.9%	1	6.7%	
Ethnicity					0.17
Not Hispanic/Latino	54	32.9%	5	33.3%	
Mexican	62	37.8%	5	33.3%	
Spanish	1	0.6%	0	–	
Central American	20	12.2%	3	20.0%	
Hispanic or Latino	10	6.1%	1	6.7%	
Latin American	7	4.3%	1	6.7%	
South American	1	0.6%	0	–	
Multiple	2	1.2%	0	–	
Not stated	7	4.3%	0	–	
BMI (kg/m^2^)	29	6.5	31	6.3	0.23
CCI score	0.93	1.4	1.3	2.0	0.44
Osteopenia	10	6.1%	0	–	1.00
Osteoporosis	1	0.6%	0	–	1.00
Corticosteroid use	3	1.8%	0	–	1.00
Alcohol abuse					0.22
Former	4	2.4%	0	–	
Active	20	12.2%	0	–	
Smoking					0.20
Former	10	6.1%	1	6.7%	
Active	46	28.0%	6	40.0%	
Homeless	5	3.0%	0	–	1.00
AO/OTA classification					0.16
41B1	4	2.4%	0	0.0%	
41B3	88	53.7%	9	60.0%	
41C1	12	7.3%	1	6.7%	
41C2	19	11.6%	1	6.7%	
41C3	41	25.0%	4	26.7%	
Schatzker Classification					0.24
I	1	0.6%	0	0.0%	
II	69	42.1%	9	60.0%	
IV	21	12.8%	0	0.0%	
V	15	9.1%	2	13.3%	
VI	58	35.4%	4	26.7%	
Medial coronal fracture	41	25.0%	6	40.0%	1
Meniscus repair	43	26.2%	8	53.3%	1
Days to surgery	17	9.3	36	67	0.28
Weight bearing compliance	160	97.6%	15	100.0%	1
Follow-up (days)	208	242	306	374	0.34

BMI, body mass index; kg, kilograms; m, meters; CCI, Charlson comorbidity index; AO/OTA, arbeitsgemeinschaft für osteosynthesefragen/orthopaedic Trauma association

Patients with rafting wires experienced significantly less linear articular subsidence (0.3 mm [95% CI − 0.3 to 0.9 mm] vs 1.0 mm [− 0.9 to 2.9 mm], *p* < 0.001) than patients without rafting wires on bivariable analysis. No patients who received rafting wires as definitive implants experienced linear subsidence ≥ 2 mm, while 22 patients (13.4%) who did not receive rafting wires experienced linear subsidence ≥ 2 mm (*p* = 0.130). No patient who received rafting wires underwent reoperation for loose or prominent implants. No erosion of rafting wires into the knee joint was observed. Multivariable linear regression demonstrated that subchondral rafting wires were the strongest (negative) predictor of linear subsidence (*β* =  − 0.596, *p* < 0.001; Table 2). 22% of variance was accounted for by the following variables: rafting wires, male sex, active smoking, and depressed plateau area.

**Table 2:** Multivariable linear regression analysis of normalized log (linear subsidence) with patient and injury characteristics

Characteristic	*β* coefficient	95% CI
Retained K-Wires	− 0.596***	− 0.847 to − 0.344
Male sex	0.214**	0.0532–0.375
Smoker		
Former	0.147	− 0.150 to 0.443
Current	0.200*	0.0441–0.356
Depressed area	0.0004*	0.0000–0.0008
Constant	− 0.615 (0.0931)	
Observations	179	
*R*^2^	0.219	
Adjusted* R*^2^	0.197	
*F* Statistic	9.71*** (df = 5; 173)	

CI, confidence interval

*p < 0.05; **p < 0.01; ***p < 0.001

## Discussion

In this multicenter retrospective cohort study, subchondral rafting wires were infrequently used as definitive implants in 8% of patients. Patients treated with rafting wires above their definitive plate and screw implants experienced statistically significant less linear fracture subsidence compared to patients without rafting wires. No patients treated with rafting wires experienced fracture subsidence ≥ 2 mm, reoperation for loose or prominent wires, or erosion of the wires into the knee joint. Multivariable regression to mitigate potential confounding demonstrated that subchondral rafting wires were the strongest negative predictor of linear subsidence (*β* =  − 1.4, *p* < 0.01).

Incongruity of the articular surface is a well-known risk factor for post-traumatic osteoarthritis and consequent pain, stiffness, and disability [18]. Parkkinen et al. reported that postoperative articular depression > 2 mm predicted severe post-traumatic arthritis in tibial plateau fractures [19]. Singleton et al. found that patients with < 2.5 mm of residual articular depression had significantly smaller losses in knee range of motion and better functional outcomes [20]. On bivariable analysis, we did not identify a statistically significant difference in the incidence of clinically relevant linear subsidence ≥ 2 mm between patients who did or did not receive rafting wires (0% vs .13.4%, *p* = 0.130). We did observe an average absolute difference in linear subsidence of 0.7 mm attributable to rafting wires. This small difference may not be clinically relevant to knee function or risk of post-traumatic arthritis, e.g. the average patient may not experience clinical benefit from an absolute decrease in linear subsidence of < 1 mm.

Subchondral metal implant rafting constructs including Kirschner wires, lag, and positional screws independent of a plate, or cortical and locking screws associated with a plate are described strategies to prevent loss of reduction at the articular surface after open reduction and internal fixation of tibial plateau fractures [11, 21]. Cole described supporting comminuted, unstable osteochondral fragments with a raft of parallel smaller-diameter screws inferior and parallel to the articular surface [22]. This strategy is supported by biomechanical research. Karunakar et al. demonstrated in a cadaveric biomechanical study that fixation constructs with a raft of subchondral screws were more resistant to local depression loads [23]. Patil et al. showed that a raft of four 3.5 mm cortical screws was biomechanically stronger than two 6.5 mm cancellous screws in resisting axial compression in osteoporotic bone and underwent less fragmentation in normal density bone [24]. Beris et al. reported that subchondral K-wire reinforcement of tibial plateau depressions significantly enhanced load tolerance in a cadaveric model [25].

The clinical value of subchondral rafting implants for tibial plateau fracture fixation has not previously been established in comparative studies. Kayali et al. observed no significant difference with respect to mechanical axis deviation, medial proximal tibia angle, and tibial slope between injured and uninjured knees of patients when 5 mm raft screws were used to support the articular reduction after internal fixation with a locking plate for the treatment of Schatzker type II fractures, but no comparator was considered [11]. Reul et al. showed successful reduction and full range of motion in 10 out of 17 patients treated with free 2.7 mm subchondral screws above a locking compression plate (LCP), without comparison to patients treated solely with a LCP [26]. Karunakar et al. supported the use of a raft construct when a tibial plateau fracture has a significant central depression component [23]. Morochovic et al. confirmed this by demonstrating that age and lateral plateau area in split-depression lateral plateau tibial fracture treated with a raft construct through a locking plate are risk factors for increased postoperative subsidence [5]. To our knowledge, this is the first comparison of radiographic outcomes of tibial plateau fractures treated with subchondral rafting wires.

Multivariable linear regression confirmed the effect of subchondral rafting wires on subsidence while introducing other predictors. We observed that the AO-OTA 41B1.3 fracture pattern, e.g. medial extension of a lateral tibial plateau fracture involving the tibial spines, conferred a statistically significant predictor of increased varus deformity. We also observed that osteoporosis predicted subsidence, consistent with observations by Patil et al. that decreased force is required to produce a 5 mm depression in an osteoporotic bone model compared to a normal bone density model [24].

There are limitations to this investigation inherent to a retrospective cohort including selection bias, confounding by indication, variation in radiologic and surgical methods, loss to follow-up, and unequal follow-up between groups. The measurement of subsidence may be sensitive to the quality of radiographic technique, particularly variation in cranial or caudal tilt of the c-arm. Radiographic technique is a probable random source of error in these measurements, decreasing the confidence in our estimates without systematic bias.

Although the treatment was rare, our sample was sufficiently powered to observe significant differences in the primary outcome measures. This work is strengthened by radiographic validation of inclusion criteria, use of prospectively collected registries, radiographic images obtained using standardized protocols, quantitative objective outcome measurements obtained with precise radiologic software, and multivariable analysis to address potential confounding.

In conclusion, subchondral rafting wires are an effective technique for reducing linear subsidence after internal fixation of tibial plateau fractures. While the average effect size is unlikely to be clinically significant, the absolute reduction in risk of subsidence ≥ 2 mm as well as low number needed to treat indicate that rafting wires as definitive implants may be useful for patients and fractures at high risk of postoperative subsidence.
